# The eukaryotic translation initiation factor 3 subunit L protein interacts with *Flavivirus* NS5 and may modulate yellow fever virus replication

**DOI:** 10.1186/1743-422X-10-205

**Published:** 2013-06-22

**Authors:** Ana TS Morais, Ana CB Terzian, Danilo VB Duarte, Roberta VM Bronzoni, Maria CFS Madrid, Arieli F Gavioli, Laura HVG Gil, Amanda G Oliveira, Cleslei F Zanelli, Sandro R Valentini, Paula Rahal, Mauricio L Nogueira

**Affiliations:** 1 Laboratório de Pesquisas em Virologia, Departamento de Doenças Dermatológicas, Infecciosas e Parasitárias, Faculdade de Medicina de São José do Rio Preto-FAMERP, Av. Brigadeiro Faria Lima 5416, São José do Rio Preto, SP 15090-000, Brazil; 2 Programa de Pós-graduação em Microbiologia, Departamento de Biologia, Universidade Estadual Paulista “Julio de Mesquita Filho”, Campus São José do Rio Preto – IBILCE/UNESP, 15054-000, São José do Rio Preto, SP, Brazil; 3 Departamento de Virologia e Terapia Experimental, Centro de Pesquisas Aggeu Magalhães CPqAM / FIOCRUZ, Av. Professor Moraes Rego s/n, Recife, PE 50670-420, Brazil; 4 Departamento de Ciências Biológicas, Faculdade de Ciências Farmacêuticas, Universidade Estadual Paulista, Araraquara 14801-902, Brazil; 5 Center for Tropical Diseases, University of Texas Medical Branch, Galveston, TX, USA

**Keywords:** Eukaryotic translation initiation factor 3 subunit L, YFV protein nonstructural NS5, Protein-protein interaction

## Abstract

**Background:**

Yellow fever virus (YFV) belongs to the *Flavivirus* genus and causes an important disease. An alarming resurgence of viral circulation and the expansion of YFV-endemic zones have been detected in Africa and South America in recent years. NS5 is a viral protein that contains methyltransferase and RNA-dependent RNA polymerase (RdRp) domains, which are essential for viral replication, and the interactions between NS5 and cellular proteins have been studied to better understand viral replication. The aim of this study was to characterize the interaction of the NS5 protein with eukaryotic translation initiation factor 3 subunit L (eIF3L) and to evaluate the role of eIF3L in yellow fever replication.

**Methods:**

To identify interactions of YFV NS5 with cellular proteins, we performed a two-hybrid screen using the YFV NS5 RdRp domain as bait with a human cDNA library, and RNApol deletion mutants were generated and analyzed using the two-hybrid system for mapping the interactions. The RNApol region involved was segmented into three fragments and analyzed using an eIF3L-expressing yeast strain. To map the NS5 residues that are critical for the interactions, we performed site-direct mutagenesis in segment 3 of the interaction domain (ID) and confirmed the interaction using *in vitro* assays and *in vivo* coimmunoprecipitation. The significance of eIF3L for YFV replication was investigated using eIF3L overexpression and RNA interference.

**Results:**

In this work, we describe and characterize the interaction of NS5 with the translation factor eIF3L. The interaction between NS5 and eIF3L was confirmed using *in vitro* binding and *in vivo* coimmunoprecipitation assays. This interaction occurs at a region (the interaction domain of the RNApol domain) that is conserved in several flaviviruses and that is, therefore, likely to be relevant to the genus. eIF3L overexpression and plaque reduction assays showed a slight effect on YFV replication, indicating that the interaction of eIF3L with YFV NS5 may play a role in YFV replication.

**Conclusions:**

Although the precise function of eIF3L on interactions with viral proteins is not entirely understood, these results indicate an interaction of eIF3L with YF NS5 and that eIF3L overexpression facilitates translation, which has potential implications for virus replication.

## Background

Yellow fever virus (YFV) is a mosquito-borne member of the *Flavivirus* genus of the Flaviviridae family. YFV infects humans and nonhuman primates and is a highly pathogenic human virus [1,2]. Severe YF is a systemic viral disease, presenting viremia, fever, prostration, hepatic, renal, and myocardial injury, hemorrhage, and shock; 20 to 50% lethality is reported for severe cases. An alarming resurgence of viral circulation and the expansion of YFV-endemic zones have been detected in Africa and South America in recent years, potentially leading to the outbreak of urban epidemics [3]. Despite the existence of an efficient vaccine, there is no antiviral drug to treat yellow fever or other flaviviruses.

In most cases, the treatment of severe YF by supportive care is essentially ineffective, and there is a clear need for safe and effective drugs to treat patients during all stages of this disease. Therefore, the target-based design of inhibitors of *Flavivirus* replication may be a promising strategy toward the development of selective anti-flaviviral drugs [2,4].

The *Flavivirus* genome consists of an 11-kb positive-strand RNA with 5′ and 3′ untranslated regions (UTRs) and a single long open reading frame (ORF). Flaviviruses produce a subgenomic, noncoding RNA (approximately 0.5 kb) that is derived from the 3′ UTR of the genomic RNA (gRNA). The subgenomic *Flavivirus* RNA (sfRNA) is a product of the incomplete degradation of gRNA, which may involve cellular 5′-3′ exoribonuclease 1 (XRN1), a key enzyme in the cellular mRNA decay pathway. sfRNAs are involved in viral replication, cytopathicity, and pathogenicity and may play a role in modulating host antiviral responses via RNA-mediated pathways [5].

The translation of the *Flavivirus* gRNA produces a polyprotein that is cleaved co- and post-translationally by a combination of host and viral proteases into three structural proteins that constitute the viral particle (capsid, C; membrane precursor, prM; and envelope, E), and seven nonstructural (NS) proteins (NS1, NS2A, NS2B, NS3, NS4A, NS4B, and NS5), which are involved in viral RNA replication, virus assembly, and the modulation of host cell responses [2,4]. NS5 is the largest and most highly conserved protein and contains two distinct enzymatic activities that are essential during viral replication. Separated by an interdomain region, the S-adenosyl methyltransferase activity is located at the N-terminus and is responsible for capping the nascent RNA, and the RNA-dependent RNA polymerase (RdRp) activity is found at the C-terminus and is responsible for replicating the viral RNA genome [3,6].

Flaviviruses enter their target cells by receptor-mediated endocytosis, whereby the acidic environment triggers major conformational changes in their envelope glycoprotein (E), inducing the fusion of the viral and host cell membranes. The RNA released into the cell encodes a polyprotein precursor that is processed as described above. During these processes, the (+) ssRNA viral genome acts as a template for (i) the synthesis of the intermediate (−) ssRNA strand by NS5 RdRp, which in turn acts as template solely for the synthesis of (+) ssRNA genomic RNAs (again by the NS5), and (ii) the synthesis of the viral polyprotein. Immature, noninfectious virions assemble within the endoplasmic reticulum (ER): the viral RNA is complexed with the C protein and packaged into an ER-derived lipid bilayer containing heterodimers of the prM and E proteins. After transport through the host secretory pathway and virion maturation in the Golgi, mature infectious particles are then released by exocytosis [4,7].

Numerous host gene products and pathways have recently been implicated in the replicative cycle of flaviviruses; however, the biological relevance of many of these interactions is not clear [7]. Indeed, the detailed analysis of these interactions might provide a better understanding of viral replication and lead to the rational development of drugs [8,9].

The 67-kDa L subunit of human eukaryotic translation initiation factor 3 (eIF3L) is one of the subunits of the mammalian translation initiation factor eIF3 [10]. The eIF3 factor plays a central role in translation initiation by recruiting the 40S ribosomal subunit and keeping it dissociated from the 60S subunit and by promoting the association of the former with mRNA and initiator Met-tRNA [10]. In addition, eIF3 interacts with other initiation factors involved in start codon selection; however, the molecular mechanisms by which eIF3 exerts these functions are poorly understood [11]. Masutani and colleagues (2007) demonstrated that the L subunit of eIF3 is dispensable in the formation of active eIF3 complexes during ribosomal recruitment, suggesting that eIF3L likely increases the physical stability of the eIF3 complex by providing intersubunit connections [12]. Furthermore, Seither and colleagues (2001) reported that eIF3L functions as an accessory protein of cellular Pol I (PAF67), suggesting that the association of eIF3L with the enzyme endows Pol I with the capability to assemble into a productive transcription initiation complex at the rDNA promoter. Despite these findings, the function of eIF3L in viral replication is unknown.

In this work, we describe and characterize the interaction of NS5 with the translation factor eIF3L, as confirmed by *in vitro* binding and *in vivo* coimmunoprecipitation assays. This interaction occurs in a region (the interaction domain of RNApol domain) that is conserved in several flaviviruses and that is, therefore, likely to be relevant to the genus. Although eIF3L overexpression and plaque reduction assays (using an YFV replicon [13]) demonstrated a slight (if any) inhibition of YFV replication, we were able to demonstrate that eIF3L overexpression facilitates translation, with potential implications in viral replication.

## Results

### Identification of cellular proteins interacting with YFV NS5

To identify cellular proteins interacting with YFV NS5, a yeast two-hybrid screen was performed using the RNA polymerase domain of NS5 (amino acids 300 to 905) as bait against a HeLa cell cDNA library. Thirty-five positive clones were obtained from 3.8 × 10^4^ independent yeast colonies. DNA sequence analysis and the BLASTN algorithm were employed to analyze the sequence data, resulting in the identification of eIF3L as a YFV NS5-interacting protein, which was detected in 3 of the 35 sequenced clones.

### Confirmation of the interaction between the RNApol domain of NS5 and eIF3L

**Figure 1:**
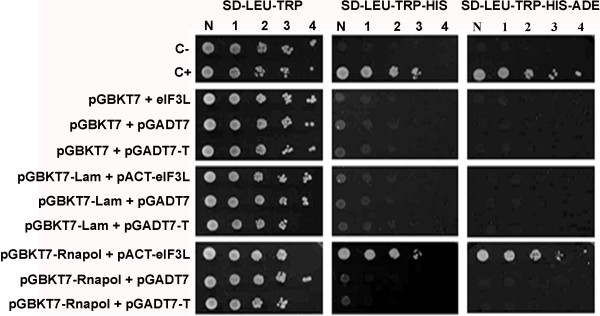
**YFV NS5(RNApol)-human eIF3L interaction in a yeast two**-**hybrid system.** Yeast strain AH109 was co-transformed with pGBKT7-RNApol (or the empty or Lamin C human protein BD vector) and pACT2-eIF3L (or the empty or SV40 large T-antigen AD vector). The specific interaction was identified by the growth of transformants on SD medium (−LEU, -TRP, -HIS) and SD medium (−LEU, -TRP, -HIS, -ADE) with the activation of the reporter genes *HIS*3 and *ADE*2. C-, negative control; C+, positive control; N, normalized original culture sample; 1–4, 10-fold serial dilutions of sample.

To confirm the interaction of the RNApol domain with eIF3L and to exclude the possibility of false-positive clones, plasmids pGBKT7-RNApol and pACT2-eIF3L were cotransfected into yeast and selected by the activation of the reporters *HIS*3 and *ADE*2. The transformants were selected on SD medium (−His, -Leu, and -Trp) and confirmed on SD medium (−Ade, -His, -Leu, and -Trp). The co-transformants grew on control drop-out medium (SD) plates lacking leucine (−Leu) and tryptophan (−Trp), as shown in Figure 1. The growth on plates lacking histidine (−His) and adenine (−Ade) was indicative of a positive interaction, as shown between the RNApol domain and eIF3L.

### Mapping the eIF3L-binding domain of RNApol

**Figure 2:**
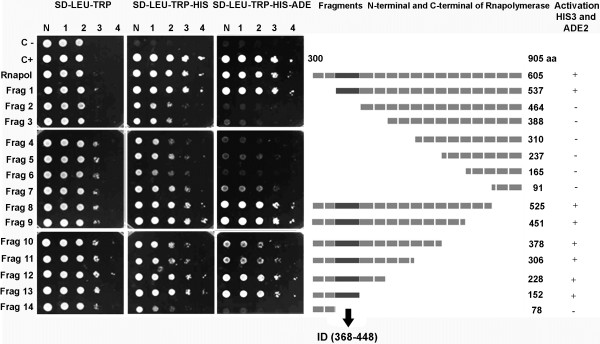
**Determination of the YFV NS5 domain responsible for the interaction with eIF3L.** The figure shows a representation of RNApol NS5 YFV and its fragments, with deletions (1–14) used to map the interaction domain with the eIF3L protein in the yeast two-hybrid system. The yeast was co-transformed with the pGBKT7-deletion constructs and pACT2-eIF3L. The interaction occurs between amino acids 368 and 448 (interaction domain, the region in black in the schematic representation), which was identified by the growth of transformants on SD medium (−LEU, -TRP, -HIS) and SD medium (−LEU, -TRP, -HIS, -ADE) with the activation of the reporter genes *HIS*3 and *ADE*2. C-, negative control; C+, positive control; N, normalized original culture sample; 1–4, 10-fold serial dilutions of sample.

A series of RNApol deletions were generated to map the interaction between the RNApol domain and eIF3L (Figure 2), and these RNApol variants were tested for interactions with eIF3L by the activation of *HIS*3 and *ADE*2 in yeast. As shown in Figure 2, the RNApol deletion mutants Δ300-368, Δ376-905, Δ450-905, Δ525-905, Δ600-905, Δ675-905, and Δ751-905 interacted with eIF3L, indicating that the interaction occurs within an 80-amino acid region (368 to 448) of RNApol known as the interaction domain (ID).

### The terminal region of the interaction domain is essential for the interaction with eIF3L

**Figure 3:**
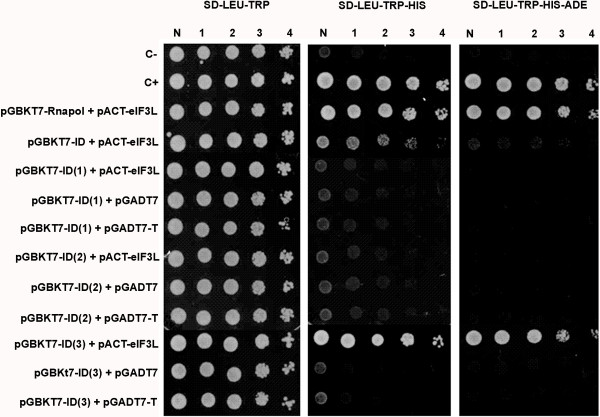
**The eIF3L protein specifically interacts with the N**-**terminal region of the NS5 YFV interaction domain.** The yeast was co-transformed with the pGBKT7 segments and pACT2-eIF3L (or the empty or large T-antigen SV40 AD vector). Segment 3 NS5 (ID) and eIF3L interaction was detected by activation of the reporter genes *HIS*3 and *ADE*2, which led to the growth of transformants on SD medium (−LEU, -TRP, -HIS) and SD medium (−LEU, -TRP, -HIS, -ADE). C-, negative control; C+, positive control; N, normalized original culture sample; 1–4, 10-fold serial dilutions of sample.

For a more precise mapping of the interaction, the interaction domain was divided into three fragments: ID1 (368 to 412), ID2 (385 to 431), and ID3 (431 to 448). The fragments were cloned and transformed into yeast along with eIF3L. As shown in Figure 3, a positive interaction occurs between the N-terminal third portion (approximately 17 amino acids) of ID and eIF3L.

### Mapping the residues of the terminal region of the interaction domain that are critical for the interaction with eIF3L

Given the previous identification of segment 3 of the ID as the site of interaction with eIF3L, this region was subjected to site-direct mutagenesis to identify the NS5 residues that are critical for the observed interaction.

**Figure 4:**
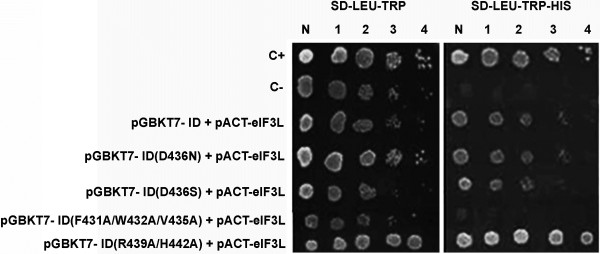
**The mapping of the terminal region residues of the interaction domain using the yeast two**-**hybrid system indicates a critical interaction with eIF3L.** The yeast was co-transformed with the mutant pGBKT7 constructs and pACT2-eIF3L. A positive interaction is shown by the activation of the reporter gene *HIS3* in the interaction of eIF3L with the mutants ID (D436N), ID (D436S), and ID (R439A/H442A), indicating that the mutations are not critical for the interaction. A negative interaction between mutant ID (F431A/W432A/V435A) and eIF3L is indicated by the absence of growth on plates lacking histidine (−His). C-, negative control; C+, positive control; N, normalized original culture sample; 1–4, 10-fold serial dilutions of sample.

Mutants ID (F431A/W432A/V435A), ID (D436N), ID (D436S), and ID (R439A/H442A) were tested for interactions with eIF3L, as described above. Yeast cells expressing mutants ID (D436N), ID (D436S), and ID (R439A/H442A) interacted with eIF3L and are, therefore, not likely to be critical for the interaction. In contrast, no growth of yeast containing the mutant ID (F431A/W432A/V435A) and eIF3L was observed on plates lacking histidine (−His) (Figure 4). This triple alanine-substitution mutation is within the FWELV region of ID, indicating that the FWELV region may be critical for the interaction with eIF3L.

### Interaction of eIF3L with the interaction domains of other flaviviruses

**Figure 5:**
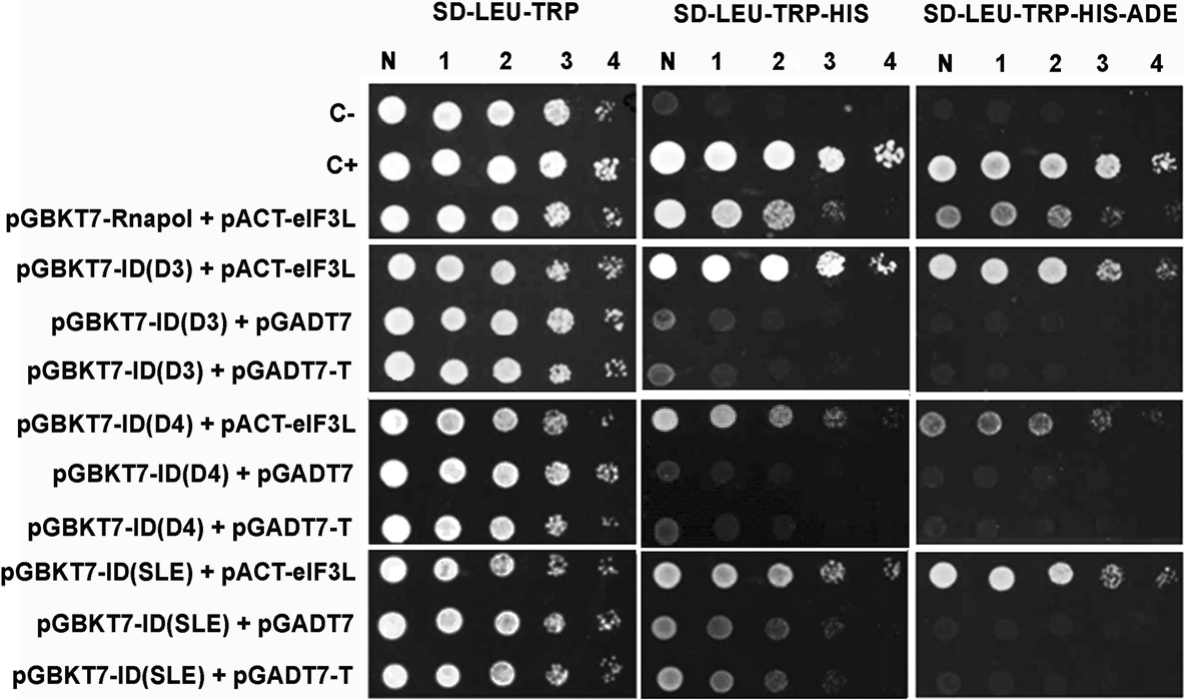
**The interaction domains of other flaviviruses interact with eIF3L.** The yeast cells were co-transformed with pGBKT7-ID (*Flavivirus* members dengue virus types 3 and 4 and St. Louis encephalitis) and pACT2-eIF3L (or the empty or large T-antigen SV40 AD vector). The interaction domains of other flaviviruses (DENV types 3 and 4 and SLE) present a positive interaction with the eIF3L protein through the activation of the reporter genes *HIS*3 and *ADE*2. C-, negative control; C+, positive control; N, normalized original culture sample; 1–4, 10-fold serial dilutions of sample.

To assess the potential interaction of eIF3L with other flaviviruses, the ID homolog from dengue virus 4, dengue virus 3, and Saint Louis encephalitis (SLE) virus were tested using the two-hybrid system. As positive interactions for pGBKT7-ID (D3), pGBKT7-ID (D4), and pGBKT7-ID (SLE) with eIF3L were demonstrated by growth on plates lacking histidine (−His) and adenine (−Ade) (Figure 5), the IDs from dengue virus 4, dengue virus 3, and Saint Louis encephalitis virus also interact with eIF3L.

### YFV NS5 (ID) binds to eIF3L *in vitro*

**Figure 6:**
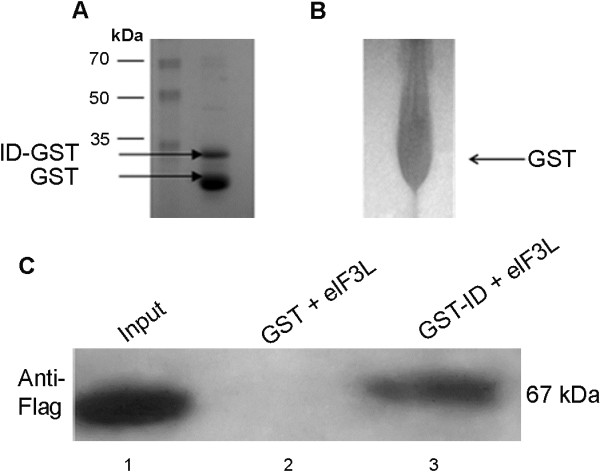
***In vitro *analyses of the YFV NS5(ID)-eIF3L interaction.** (**A**) pGEX 5X1-ID plasmid, the GST-fused ID (33 kDa) expressed in *E*. *coli* BL21 (DE3). (**B**) Control GST (26 kDa) expressed in *E*. *coli* BL21 (DE3). (**C**) HeLa cells were transfected with a plasmid expressing Flag-tagged eIF3L (67 kDa) (column 1). This protein was incubated with resin-bound GST or GST-ID. After the samples were washed, the resin-bound proteins were resolved by SDS-PAGE and then analyzed by a western blot assay using an anti-Flag antibody. The interaction of Flag-eIF3L with GST-ID (column 3) was confirmed, without a nonspecific interaction with GST (column 2).

The interaction between the YFV NS5 interaction domain and eIF3L was confirmed using a glutathione S-transferase (GST) pull-down assay. These assays were performed using purified GST-ID and GST bound to glutathione-Sepharose beads in combination with Flag-tagged eIF3L produced in HeLa cells. As shown in Figure 6, Flag-eIF3L interacted with the GST-ID fusion protein (Figure 6, column 3) but not with the negative GST control (Figure 6, column 2).

### YFV NS5 interacts with eIF3L in mammalian cells

**Figure 7:**
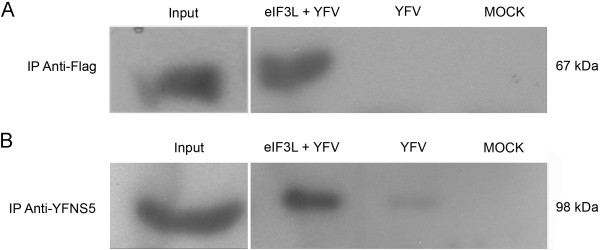
**Coimmunoprecipitation of YFV NS5 and eIF3L in mammalian cells.** Vero cells were transfected with a plasmid expressing Flag-tagged eIF3L and infected with YFV strain 17D at an M.O.I. of 5. At 48 h post-infection, the cells were lysed and subjected to immunoprecipitation using anti-Flag M2 affinity gel beads. The coimmunoprecipitated proteins were analyzed by a western blot assay using an anti-YFNS5 antibody. (**A**) Cell lysates immunoprecipitated using the anti-Flag antibody. Input, positive control eIF3L (67 kDa) from transfected cells; eIF3L+YFV, eIF3L immunoprecipitated from transfected and infected cells; YFV, infected and non-transfected cells; MOCK, non-infected and non-transfected cells. (**B**) Coimmunoprecipitation of the NS5 protein with the anti-YFV NS5 antibody. Input, positive control YFV NS5 from infected cells (98 kDa); eIF3L+YFV, coimmunoprecipitation of YFV NS5 with Flag-eIF3L showing a positive interaction; YFV, MOCK, YFV NS5 was not precipitated in non-transfected cells.

Coimmunoprecipitation assays were performed to confirm that the YFV NS5 protein interacts with eIF3L in mammalian cells. Vero cells were transfected with Flag-eIF3L and infected with YFV. Cell lysates containing both NS5 and Flag-eIF3L were incubated with anti-Flag beads, and the eluates were subjected to western blot analyses. As shown in Figure 7B, YFV NS5 was efficiently coimmunoprecipitated with Flag-eIF3L. In contrast, YFV NS5 was not precipitated in the absence of Flag-eIF3L. Similarly, no YFV NS5-reactive species were detected in the cell lysates containing only the Flag-eIF3L protein. These results indicate that YFV NS5 can interact with eIF3L *in vivo*.

### The potential role of eIF3L in yellow fever virus replication

To establish the significance of these interactions within a physiological context, we evaluated the role of eIF3L using overexpression and RNA interference assays.

**Figure 8:**
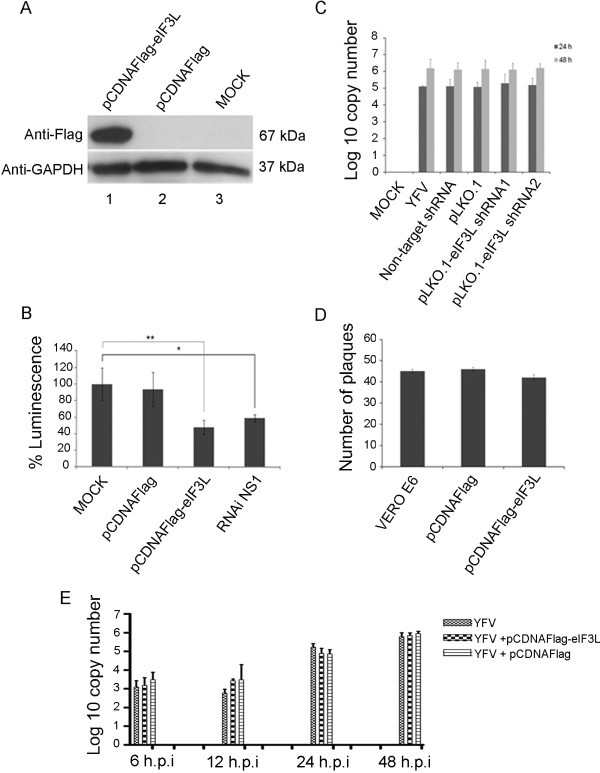
**Effect of eIF3L on yellow fever virus replication.** (**A**) eIF3L overexpression in BHK21-rep-YFV17D LucNeoIres cells at 48 h post-transfection using an anti-Flag antibody or anti-GAPDH antibody as a control. eIF-pCDNAFlag, overexpressing eIF3L; pCDNAFlag, empty vector control; MOCK, control cells. (**B**) Luciferase activity at 48 h transfection with eIF3L in BHK21-rep-YFV17D LucNeoIres cell lysates; MOCK, control cells; pCDNAFlag, empty vector control; eIF3L-pCDNAFlag, eIF3L overexpression; siRNA NS1, positive control. *Significantly different between the RNAi NS1 and mock samples; **significantly different between the pCDNAFlag-eIF3L and mock samples. (**C**) Quantification of viral RNA transcripts at 24 and 48 h post-infection with pseudotyped lentiviral particles using real-time PCR analysis. pLKO.1-eIF3LshRNA1, eIF3L silenced and infected cells; pLKO.1-eIF3LshRNA2, eIF3L silenced and infected cells; pLKO, empty vector control-infected cells; non-target, plasmid control non-target infected cells; YFV, infected cells; MOCK, control cells. (**D**) The plaque reduction assay indicates a discrete reduction in viral replication. (**E**) Quantification of viral RNA in Vero cell supernatants overexpressing eIF3L at 6, 12, 24, and 48 h post-infection.

For overexpression, BHK-21-Rep-YFV17D LucNeoIres cells, a stable bicistronic dual-reporter YFV replicon cell line expressing the neomycin phosphotransferase resistance gene and the reporter gene firefly luciferase [13], were used. The cells were transfected with the pCDNAFlag-eIF3L plasmid or an empty vector. After 48 h, the cells were collected for a western blot analysis and luciferase activity assays. eIF3L overexpression relative to cells transfected with the empty vector or to untransfected cells is shown in Figure 8A (column 1). The levels of luciferase activity in the cell lysates were significantly reduced by 50% compared to the controls (Student’s *t*-test; p<0.05) (Fig. 8B). An shRNA against YF [14] was used as a positive control.

Furthermore, eIF3L expression was downregulated in HEK293T cells infected with pseudotyped lentiviral particles containing specific pLKO.1-eIF3LshRNA1 or pLKO.1-eIF3LshRNA2 or negative controls. In the silenced cells, the levels of viral RNA (as determined by real-time PCR) were not significantly reduced in comparison to the controls (Figure 8C).

We also overexpressed eIF3L in Vero cells and infected the cells with 50 PFU of YFV17D virus. At 48 h after infection, a slight inhibition of yellow fever virus replication (but without statistical significance) was observed in the pCDNAFlag-eIF3L-transfected cells compared to the controls (Figure 8D). In addition, we also overexpressed eIF3L in Vero cells and infected them with a multiplicity of infection (M.O.I.) of 1 for the YFV 17D virus. Supernatants were collected at 6, 12, 24, and 48 h.p.i., and qRT-PCR was performed. Similar to the above results, the levels of viral RNA were not significantly reduced in the pCDNAFlag-eIF3Ltransfected cells (Figure 8E).

**Figure 9:**
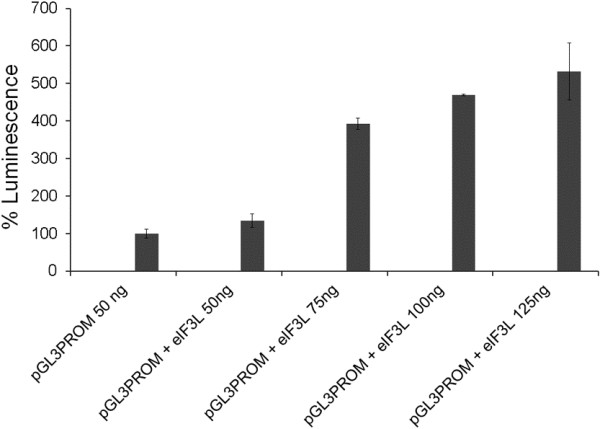
**Effect of eIF3L expression on general translation.** The HeLa cells were cotransfected with pCDNAFlag-eIF3L and pGL3 promoter plasmids in a dose-dependent manner. After 48 h, the cells were lysed, and the supernatant was used for luciferase activity assays.

As it can be argued that the addition of eIF3L can inhibit translation by itself (i.e., as very little is know regarding the physiological role of eIF3L in the translation complex), we also verified the effect of eIF3L expression on general translation. Thus, the plasmid pGL3Promoter (Promega) was co-transfected into HeLa cells with eIF3L in a dose-dependent manner. As can be observed in Figure 9, eIF3L in fact facilitated translation in the same amount that inhibited the luciferase-YFV replicon.

## Discussion

Although numerous host gene products and pathways have recently been implicated in the replicative cycle of flaviviruses, their biological relevance remains unclear [7]. Based on previous demonstrations of interactions of the NS5 proteins of other *Flavivirus* with host cellular proteins [15-22], in the present study, we analyzed YFV NS5-RdRp for its role in viral replication by identifying host proteins that interact with the YFV NS5 RdRp domain and assessing their functions in viral replication. These experiments were performed only with YFV NS5 RdRp because the methyltransferase domain is likely responsible for the cytotoxic effect that we observed in yeast (data not shown).

Using yeast two-hybrid, *in vitro* binding, and coimmunoprecipitation assays, we demonstrated that the YFV NS5-RdRp protein interacts with eukaryotic translation initiation factor 3 subunit L (eIF3L). Although eIF3L has an essential cellular function, its significance in viral replication remains to be determined, and even its cellular role is uncharacterized.

During viral replication, the virus enters the cell, the nucleocapsid disassembles, and replication then proceeds with the immediate translation of the genome. The 5′-end of the (+) ssRNA genome is decorated by an RNA cap structure (N7meGpppA2’Ome-RNA) that plays an essential role in the initiation of translation and in protecting the viral RNA from degradation by endogenous RNA exonucleases [4]. The virus presumably borrows host eukaryotic translation initiation factors (eIFs) to initiate translation [3]. Indeed, previous studies have demonstrated the association of eIFs, including eIF3, in viral replication [23-25] and have also reported the involvement of other proteins, such as eIF3F, with this complex to inhibit viral replication [24].

Schoggins et al. (2011) used a screening approach along with eIF3L overexpression to demonstrate a modest inhibitory potential (approximately 10%) on YFV replication [26]. Within a physiological context, eIF3L expression is downregulated in peripheral blood mononuclear cells after vaccination with the yellow fever 17D virus [27]. These findings suggest that, after entry of the virus into the cells, YFV can induce the downregulation of eIF3L as a way to escape the host’s immune system.

In this study, we have demonstrated that eIF3L overexpression in mammalian cells results in a significant decrease in luciferase activity in assays. However, we used a cell lineage carrying a yellow fever virus replicon with two different points of translation, a 5′ cap end and an internal ribosome entry site (IRES) element; eIF3 complex proteins were involved in their translation in both cases. The eIF3L protein is a subunit of the eIF3 translation complex, and its overexpression could affect replicon translation, with a downregulation of luciferase activity though without inhibiting viral replication. Therefore, to determine the direct effect of eIF3L on replication, a plaque reduction assay and quantification of viral RNA after overexpressing or silencing eIF3L were performed. Although the levels of viral RNA were not significantly reduced in the silenced cells, the plaque reduction assay and qRT-PCR after overexpression displayed modest levels of inhibition (but not statistically significant) on viral replication. It is important to note that eIF3L overexpression did not affect the global synthesis of proteins in the cell, which was shown by the endogenous control of GAPDH, and that the same concentration that facilitated translation inhibited yellow fever virus replication (Figure 9).

Therefore, our results are in agreement with the findings in the literature, though further studies will be necessary to understand the mechanisms involved in viral replication.

Soon after translation, NS5 RdRp initiates the synthesis of full-length negative-sense RNA copies from the genome template that are transcribed to produce progeny positive-sense RNA genomes [3]. Mammalian RNA polymerase I (Pol I) is a multisubunit enzyme that interacts with accessory proteins, PAFs (polymerase-associated factors), including eIF3L (PAF67), and the available data suggest the involvement of these proteins in the initiation of transcription [28]. Yuan et al. (2002) demonstrated that the transcription factor TIF-IA is strongly associated with eIF3L (PAF67); TIF-IA is also associated with a subpopulation of Pol I to form the transcriptionally active enzyme and is a target of diverse signaling pathways [29]. Therefore, eIF3L binding to TIF-IA may be a target of regulatory pathways in association with the YFV NS5 RNA polymerase.

Using two-hybrid assays, we demonstrated that the interaction occurred within an 80-amino acid region (interaction domain, ID) of RNApol. As reported previously [30], the production of a homology model for the RNA polymerase domain of the YFV NS5 protein showed that this region of interaction (ID) is exposed and potentially capable of forming interactions. We also demonstrated that ID from the dengue 4, dengue 3, and Saint Louis encephalitis viruses interact with eIF3L, suggesting that this interaction is important for other members of the genus. Accordingly, when the YFV sequence was compared to the sequences of other flaviviruses, a region of homology from residues 20 to 80 was identified [30]. Mutations in the FWELV motif of ID abolished the interaction with eIF3L, suggesting that the amino acids FWELV are critical for the ID interaction with eIF3L. The three mutations produced in this region may have led to changes in protein conformation, preventing possible interactions with other cellular proteins. However, the data from another study in our laboratory [30] have demonstrated that the same mutations do not affect the interaction of the ID domain with the cellular small ribonucleoprotein polypeptide A (U1A), indicating that the structure of the domain is not significantly altered and is potentially capable of interacting with other molecules.

## Conclusion

In summary, we identified that the eIF3L protein interacts with the yellow fever NS5 protein. Although the precise function of eIF3L in the viral life cycle is still not entirely understood and the results for viral replication are inconclusive, we were able to demonstrate that eIF3L overexpression facilitates translation and that the interaction of eIF3L with YF NS5 may have an important role in viral replication. Further studies on this interaction may lead to a better understanding of *Flavivirus* replication and pathogenicity.

## Methods

### Plasmid construction

For the yeast two-hybrid system, the plasmids pGBKT7 and pACT2 (Clontech Inc., USA) were used as the sources of the GAL4 DNA-binding domain (BD) and GAL4 transcriptional activation domain (AD), respectively. The plasmid pACNR-FLYF17D [31] contains the complete genome of YFV and was kindly provided by Charles Rice (Rockefeller University, USA). For the construction of pGBKT7-RNApol, the RNApol domain (corresponding to amino acids 300 to 905 of YFV NS5 [32]) was amplified by PCR using the plasmid pACNR-FLYF17D. Oligonucleotides 5′-TATGAATTCAATGACAACCCTACAGGACC-3′ and 5′-TATCTGCAGTCAGATAAGCTCACCCAGTTGC-3′ were used as the plus- and minus-strand DNA primers, respectively. The PCR products were digested with *EcoR*I and *Pst*I and cloned into the corresponding sites of the pGBKT7 plasmid. For mapping the interaction, pGBKT7-RNApol deletion mutants, Δ300-367, Δ300-441, Δ300-517, Δ300-595, Δ300-668, Δ300-740, Δ300-814, Δ825-905, Δ751-905, Δ676-905, Δ604-905, Δ526-905, Δ450-905, and Δ376-905, were prepared by PCR using pACNR-FLYF17D as a template and the following primers: 5′-TATGAATTCGCGGGAACTAGGAAGATC-3′ (forward, Δ300-367), 5′- TATGAATTCCACCAACAAGGCAGGTGT-3′ (forward, Δ300-441), 5′-TATGAATTCGTG ATCAGAGACCTGGCT-3′ (forward, Δ300-517), 5′-TATGAATTCCGACGAGACCAGAGA GG-3′ (forward, Δ300-595), 5′-TATGAATTCGTGGTCCGGCCCATC-3′ (forward, Δ300-668), 5′- TATGAATTCGGAAGGGTGTCTCCAGG-3′ (forward, Δ300-740), 5′-TATGAATTCGTG TGGAACAGAGTATGG-3′ (forward, Δ300-814), 5′-TATCTGCAGTCACCATACTCTGTT CCACAC-3′ (reverse, Δ825-905), 5′-TATCTGCAGTCATCCTGGAGACACCCTTCC-3′ (reverse, Δ751-905), 5′-TATCTGCAGTCAGATGGGCCGGACCAC-3′ (reverse, Δ676-905), 5′-TATCTGCAGTCATCCTCTCTGGTCTCGTCG-3′ (reverse, Δ604-905), 5′-TAT CTGCAGTCAAGCCAGGTCTCTGATCAC-3′ (reverse, Δ526-905), 5′-TATCTGCAGTCA ACACCTGCCTTGTTGGTG-3′ (reverse, Δ450-905), and 5′-TATCTGCAGTCAGATCTTCCT AGTTCCCGC-3′ (reverse, Δ376-905). The PCR products were digested with *EcoR*I and *Pst*I and cloned into the corresponding sites of the pGBKT7 plasmid. pGBKT7-ID, with an interaction domain sequence corresponding to amino acids 368 to 448 of the RNApol domain, was generated by PCR using the plasmid pACNR-FLYF17D as a template and the following primers: 5′-TATGAATTCGCGGGAACTAGGAAGATC-3′ (forward) and 5′-TATCTGCAGTCAACACCTGCCTTGTTGGTG-3′ (reverse). The PCR products were digested with *EcoR*I and *Pst*I and cloned into the corresponding sites of the pGBKT7 plasmid. For the construction of pGBKT7-ID (1) (corresponding to amino acids 368 to 412 of the interaction domain), pGBKT7-ID (2) (amino acids 385 to 431 of the interaction domain), and pGBKT7-ID (3) (amino acids 431 to 448 of the interaction domain), we performed PCR amplifications using pACNR-FLYF17D as the template and the following primers: ID (1) 5′-GCGGGAAACTAGGAAGATC-3′ (forward) and 5′-AGCTCCAATGGCTGCATGACTTCG-3′ (reverse), ID (2) 5′-CTGGCCAGAGAAAAGAAACCCC-3′ (forward) and 5′-CTTTGGGTCTTGGACAGC-3′ (reverse), ID (3) 5′-TTCTGGGAACTGGTGGATGAA-3′ (forward) and 5′-ACACCTGCCTTGTTGGTG-3′ (reverse). The PCR products were digested with *EcoR*I and *Pst*I and cloned into the corresponding sites of the pGBKT7 plasmid.

Plasmids pGBKT7-ID (D3), pGBKT7-ID (D4), and pGBKT7-ID (SLE) were generated using dengue 3 (RPDen06/41) (AY858045), dengue 4 (Boa Vista) (AY762085) and SLEV (BeH 355964) (AY632544) as the template and primers: ID (D3) 5′-TATGGATCCTATGGCTTTGGAGAACCCTGGG-3′ (forward, *BamH*I) and 5′-CTCCTGCGAACACTTGCCCAATTTGTGGAGTTC-3′ (reverse, *Pst*I); ID (D4) 5′-TATGAATTCTGGCTGTGGGCCCTCCTTGG-3′ (forward, *EcoR*I) and 5′-TTACTGCAGACATTTCCCTTCTTGGTGCAGAGCC-3′(reverse, *Pst*I); and ID (SLE) 5′-TATGGATCCTATGGCTGTGGGACTTCGTTGC-3′ (forward, *BamH*I) and 5′-CTCCTGCAAGACACTCTCCTTTAAGATGGGCTTC-3′ (reverse, *Pst*I). The PCR products were digested and cloned into the corresponding sites of the pGBKT7 plasmid.

All amplicons were sequenced using Big Dye v3.1 and an ABI3130 DNA sequencer (Applied Biosytem, Foster City, CA).

The plasmid pSGFlag-eIF3L (kindly provided by Pierre Jalinot, Laboratorie de Biologie Moleculaire de la Cellule, Lyon, France) [10] codes for the eIF3L protein and was used for the *in vitro* pull-down and *in vivo* coimmunoprecipitation assays.

The plasmids pLKO.1-eIF3L shRNA1 (5′ CCGGCCTGGTAGACAAATCCAACATCTCGAGATGTTGGATTTGTCTACCAGGTTTTTG 3′) pLKO.1-eIF3L shRNA2 (5′CCGGACACACATTCAGGAACCTGTTCTCGAGAACAGGTTCCTGAATGTGTGTTTTTTG 3′) (Sigma) (MISSION shRNA plasmid DNA, NM_016091.2-786, NM_016091.2-1791, respectively), VSVG, and pCMV R8.74 (provided by Renato Santana de Aguiar, Molecular Virology Laboratory, Rio de Janeiro, Brazil) were used in the construction of pseudotyped lentiviral particles.

The pGL3 promoter and pGL3 basic vector (provided by Thomas Khristie, Bethesda, USA), containing the gene for luciferase expression, were used for eIF3L overexpression during translation.

### Site-directed mutagenesis

Site-direct mutagenesis was performed using the Quick Change Site Direct Mutagenesis kit (Stratagene, USA) and pGBKT7-ID as the template with overlapping primers: for ID (F431A/W432A/V435A), 5′-CTGTCCAAGACCCAAAGGCCGCGGAACTGGCGGATGAAGAAAGG-3′ (forward) and 5′-CCTTTCTTCATCCGCCAGTTCCGCGGCCTTTGGGTCTTGGACAG-3′ (reverse); for ID (D436N), 5′-GTTCTGGGAACTGGTGAATGAAGAAAGGAAGCTGC-3′ (forward) and 5′-GCAGCTTCCTTTCTTCATTCACCAGTTCCCAGAAC-3′ (reverse); for ID (D436S), 5′-GTTCTGGGAACTGGTGAGTGAAGAAAGGAAGCTGCAAC-3′ (forward) and 5′-GGTGCAGCTTCCTTTCTTCACTCACCAGTTCCCAGAAC-3′ (reverse); and for ID (R439A/H442A), 5′-GGAACTGGTGGATGAAGAAGCGAAGCTGGCCCAACAAGGC-3′ (forward) and 5′-GCCTTGTTGGGCCAGCTTCGCTTCTTCATCCACCAGTTCC-3′ (reverse). The PCR products were digested with *Dpn*I to eliminate the methylated parental DNA templates, and the correct mutant clones were verified by DNA sequencing and then subcloned back into a new pGBKT7 vector.

### Yeast two-hybrid screen

The yeast strain *Saccharomyces cerevisiae* AH109 (BD MATCHMAKER GAL4 Two-Hybrid System 3, Clontech, USA) was used for the two-hybrid screen. The bait plasmid pGBKT7-RNApol and the pACT2 HeLa cell cDNA library (Clontech, USA) were sequentially introduced into the yeast cells using the EZ-Yeast Transformation kit - Single Vector Transformation protocol (Q.BIO gene, USA). Transformants were initially selected on SD medium (−His, -Leu, and -Trp) and confirmed on SD medium (−Ade, -His, -Leu, and -Trp). The SV40 large T-antigen and p53 were used as positive interaction controls, whereas Lamin C was used as a negative control. The filter lift assay was performed with isolated colonies for β-galactosidase activity. The prey plasmids were selected from yeast colonies and by providing a positive signal, according to the manufacturer’s recommendations. False positives were eliminated by retransforming the isolated plasmids into the AH109 strain containing pGBKT7-RNApol. Plasmid DNA was isolated (RPM Yeast Plasmid Isolation kit, Q.BIO gene, USA) from the positive clones and sequenced according to the manufacturer’s recommendations. To demonstrate the interaction of RNApol with eIF3L, plasmids pGBKT7-RNApol and pACT2-eIF3L were cotransfected into yeast using the lithium acetate method. Transformants were selected on SD medium (−His, -Leu, and -Trp) and confirmed on SD medium (−Ade, -His, -Leu, and -Trp).

### Interaction mapping

For mapping the interactions, pGBKT7-RNApol deletion mutants were transfected into yeast containing pACT2-eIF3L and selected as described. Conversely, the RNApol region of the interaction (the interaction domain) was divided into three fragments, and the plasmids pGBKT7-ID (1), pGBKT7-ID (2), and pGBKT7-ID (3) were transfected into yeast containing pACT2-eIF3L. Site-direct mutagenesis was performed in segment 3 of ID to map the NS5 residues that are critical for its interaction with eIF3L. Plasmids pGBKT7-ID (FWELV), pGBKT7-ID (NEE), pGBKT7-ID (SEE), and pGBKT7-ID (RKLH) were analyzed as described above. To analyze the possible interaction of eIF3L with the interaction domains of other flaviviruses, the ID from the dengue 2, dengue 3, and Saint Louis encephalitis viruses were also tested using plasmids pGBKT7-ID (D3), pGBKT7-ID (D4), and pGBKT7-ID (SLE), respectively.

### Cells and viruses

HeLa cells (human epithelial cervical cancer) were grown in Dulbecco’s Modified Eagle’s medium (DMEM) containing 10% fetal bovine serum (FBS), 100 U/ml of penicillin, and 100 U/ml of streptomycin. Vero cells (African green monkey kidney) were grown in minimum essential medium (MEM) containing 10% FBS, 100 U/ml of penicillin, and 100 U/ml of streptomycin. The BHK-21-Rep-YFV17D LucNeoIRES cell line containing the YFV bicistronic replicon (provided by Laura Gil, Aggeu Magalhães Research Center, Fiocruz, Brazil) was grown in minimum essential medium (MEM) containing 10% FBS, 100 U/ml of penicillin, 100 U/ml of streptomycin, and 500 μg/ml G418. The cells were maintained at 37°C in a humidified atmosphere containing 5% CO_2_. Yellow fever virus strain 17D (Fiocruz, Brazil) was expanded in C6/36 cells. The infected tissue culture supernatant was used as the source of virus after the determination of the viral titers.

### Recombinant protein purification

pGEX 5X1-ID and pGEX 5X1, the GST-fused ID and control GST, respectively, were expressed in *E*. *coli* BL21 (DE3) (Stratagene, USA). Briefly, 500-ml cultures were induced for 3 h with isopropyl-β-D-thiogalactopyranoside (1 mM) at 37°C, lysed in buffer (10 mM Tris–HCl [pH 8.0], 150 mM NaCl, 1 mM EDTA, 1 mM dithiothreitol, 1% Triton X-100, and 2 mM benzamidine), and sonicated 4 times (20 s each). After centrifugation, the supernatants were applied to a glutathione-Sepharose 4B column (GE Healthcare, Brazil) according to the manufacturer’s recommendations. Fractions containing GST-ID or GST were concentrated using a Centricon filter (Millipore, USA).

### *In vitro* binding assay

For the *in vitro* binding assays, 10 μg of GST or GST-ID was bound to glutathione-Sepharose 4B (50% slurry) in PBS for 1 h (4°C). After four washes with 1 ml phosphate-buffered saline (PBS) and a final wash with lysis buffer (50 mM Tris–HCl [pH 7.5], 150 mM NaCl, 0.5% NP-40, 1 mM sodium fluoride, 0.2 mM sodium orthovanadate, 10 mM β-glycerophosphate, and protease inhibitor cocktail Mini complete Roche®), 600 μg of extract derived from HeLa cells transfected with the eIF3L-pSGFlag plasmid was added and incubated for 2 h (4°C). The unbound fractions were collected, and the beads were washed four times with 1 ml of lysis buffer. The bound fraction was eluted by incubation with SDS sample buffer and separated using 10% SDS-polyacrylamide gels, followed by immunoblotting with anti-Flag antibodies (1:1000; Sigma, USA). Antibody binding was detected using the Super Signal West Dura Pico system (Pierce Biotechnology, USA) and analyzed using a Kodak Gel Logic 2200 Image Station (Kodak, USA).

### Coimmunoprecipitation

Coimmunoprecipitations were performed using anti-Flag M2 Affinity gel (Sigma, USA). Vero cells were transiently transfected with pSGFlag-eIF3L; after 24 h, the cells were infected with YFV strain 17D at an M.O.I. of 5 and incubated in DMEM medium without FBS for 2 h. The infected cells were washed with PBS and reincubated in DMEM medium supplemented with 1% FBS for 48 h in the presence of 5% CO_2._ Non-transfected and non-infected Vero cells were used as the controls. The cells were harvested in lysis buffer (50 mM Tris–HCl [pH 7.5], 150 mM NaCl, 0.1% NP-40, 5% glycerol, 1 mM sodium fluoride, 0.1 mM sodium orthovanadate, 10 mM β-glycerolphosphate, and protease inhibitor cocktail Mini complete Roche®). Equal amounts of protein extracts were subjected to immunoprecipitation for 3 h (4°C) using anti-Flag M2 Affinity gel beads. After incubation, the beads were washed four times with TBS buffer (50 mM Tris–HCl [pH 7.4] and 150 mM NaCl). The antibody-protein complexes were then resolved by SDS-PAGE, and the YFV NS5 protein was identified by western blotting using an anti-YFNS5 (1:4000) antibody, a kind gift of Charles M. Rice, Rockefeller University.

### Overexpression experiments and luciferase activity assays

BHK21-Rep YF17D LucNeoIres cells that were 70% confluent were transfected using the Lipofectamine™ 2000 reagent (Invitrogen, USA) according to the manufacturer’s recommendations. The empty vector and untransfected cells were used as the negative controls; RNAi against NS1 YFV [14] was used as a positive control. After 48 h of transfection, the cells were lysed by the addition of Cell Culture Lysis Buffer (Promega, USA), followed by incubation at room temperature for 5 min. The cell extracts were centrifuged at 12,000 × g for 15 s (room temperature). The luciferase assays were performed using 10 μl of cell supernatants mixed with 100 μl of the luciferin substrate Luciferase 1000 Assay System (Promega, USA) prior to the measurement of luciferase activity using a Sirius luminometer (Berthold, Australia). Comparisons between the three means were analyzed using Student’s *t*-test at a significance level of 5%. The cells were also harvested in lysis buffer (50 mM Tris–HCl [pH 7.4], 150 mM NaCl, 1% Triton X-100, 1 mM EDTA, 1 mM dithiothreitol, 1 mM sodium fluoride, 0.2 mM sodium orthovanadate, 10 mM β-glycerolphosphate, and protease inhibitor cocktail Mini complete Roche®) for a western blot analysis, as described above.

eIF3L overexpression was also performed in Vero cells to better examine the alterations in the kinetics of viral replication. For these assays, Vero cells were transfected with the pCDNAFlag-eIF3L plasmid or a control using the Turbofect transfection reagent (ThermoScientific) at a ratio of 1:3, essentially as described by the manufacturer. At 24 h after transfection, the cells were counted and infected with YFV 17D at an M.O.I. of 1 for 2 h (37°C). The inoculum was then removed, washed 3× with 1× PBS and replaced with maintenance medium. The supernatants were collected 6, 12, 24, and 48 h.p.i., and RNA was extracted using the TRIzol reagent (Invitrogen) as described by the manufacturer. Real-time PCR reactions were performed using YF-specific primers (5’-TTGAACTACATGAGCCCACATC-3′ (forward) and 5′-CATAAGTCACTACCTGCCCCG-3′ (reverse) and the Fast SYBR Green Master Mix (Applied Biosystems) for viral copy number quantification.

### Construction of pseudotyped lentiviral particles

For lentiviral pseudotyped particle (pp) production, pLKO.1-eIF3L shRNA1 or pLKO.1-eIF3L shRNA2 was co-transfected with the pCI-VSVG and p-CMV plasmids into HEK-293T cells. The supernatant containing the lentivirus particles was harvested to determine the viral titer. The control vector pLKO.1-puro (Cat. # SHC001, Sigma) and negative control shRNA (Cat. # SHC002, Sigma) were used as the controls.

### Knock-down experiments using shRNA and infection assays

HEK-293T cells were infected with pseudotyped lentiviral particles at an M.O.I. of 0.1 for 2 h. The infected cells were washed with PBS and incubated in DMEM medium supplemented with 1% FCS for 72 h in the presence of 5% CO_2_. The viral RNA copy number was determined using real-time PCR analysis, as described above. The cell supernatants were harvested, and RNA was isolated using the viral RNA isolation kit (HiSpeed Plasmid Midi Kit, Qiagen). The cells were also harvested in lysis buffer (50 mM Tris–HCl [pH 7.4], 150 mM NaCl, 1% Triton X-100, 1 mM EDTA, 1 mM dithiothreitol, 1 mM sodium fluoride, 0.2 mM sodium orthovanadate, 10 mM β-glycerolphosphate, and protease inhibitor cocktail Mini complete Roche®) for a western blot analysis, as described above.

### Plaque reduction assay

Vero cells were plated in 6-well microplates (4 × 10^5^ cells/well) and transfected with pCDNAFlag-eIF3L plasmid using the Lipofectamine™ 2000 reagent (Invitrogen, USA) according to the manufacturer’s recommendations. The empty vector was used as a negative control. After 24 h of transfection, the cells were infected with 50 PFU of YFV 17DD for 2 h at 37°C for adsorption. The monolayer was washed with PBS and overlaid with MEM medium + 1% carboxymethylcellulose (CMC, Sigma, Brazil) for 5 days at 37°C in the presence of 5% CO_2_. The maintenance medium was then removed, and the cells were fixed and stained with crystal violet. The plaques were counted and compared to the controls.

### Effect of eIF3L overexpression on cell translation

To determine the effect of eIF3L overexpression on translation, HeLa cells were plated in 96-well microplates (1.5 × 10^4^ cells/well) and cotransfected with pCDNAFlag-eIF3L and pGL3 promoter plasmids at different concentrations. After 48 h, the cells were lysed, and the supernatant was used for luciferase activity assays.

## Abbreviations

YF: Yellow fever; YFV: Yellow fever virus; RdRp: RNA-dependent RNA polymerase; ID: Interaction domain; RNApol: RNA polymerase; eIF3L: Eukaryotic translation initiation factor 3 subunit L; UTRs: Untranslated regions; ORF: Open reading frame; NS5: Protein nonstructural 5; sfRNA: Subgenomic RNA; gRNA: Genomic RNA; ISGs: Interferon-stimulated genes; PAFs: Polymerase-associated factors; Pol I: RNA polymerase I; D3: Dengue virus 3; D4: Dengue virus 4; SLE: Saint Louis encephalitis.

## Competing interests

The authors declare that they have no competing interests.

## Authors’ contributions

ATSM performed the plasmid construction; site-direct mutagenesis, coimmunoprecipitation, *in vitro* binding assay, overexpression and activity luciferase assays, plaque reduction assay, and effect of eIF3L overexpression on cell translation study and participated in the preparation of the manuscript. RVMB, AFG, and MCFSM performed the plasmid construction and mapped the interaction using the yeast two-hybrid system. DVBD performed the purification of the recombinant protein and participated in executing the *in vitro* and co-immunoprecipitation assays. ACBT performed the knock-down, overexpression, and infection assays. LHVGG, AGO, CFZ, and SRV contributed new analytical tools and to the experimental design. MLN, PR, and RVMB conceived the study, designed and supervised the experiments, and revised the manuscript. All authors have read and approved this manuscript.
